# Memory B cell development in response to mRNA SARS-CoV-2 and nanoparticle immunization in mice

**DOI:** 10.1073/pnas.2527869123

**Published:** 2026-01-02

**Authors:** Marie Wiatr, Zijun Wang, Marie Canis, Brianna Hernandez, Anna Gazumyan, Gabriela S. Silva Santos, Sadman Shawraz, Songhee Lee, Paul D. Bieniasz, Theodora Hatziioannou, Frauke Muecksch, Michel C. Nussenzweig

**Affiliations:** ^a^Laboratory of Molecular Immunology, The Rockefeller University, New York, NY 10065; ^b^Laboratory of Retrovirology, The Rockefeller University, New York, NY 10065; ^c^HHMI, The Rockefeller University, New York, NY 10065; ^d^Department of Infectious Diseases, Virology, Medical Faculty Heidelberg, Heidelberg University, Heidelberg 69120, Germany

**Keywords:** nanoparticle vaccine, mRNA vaccine, memory B cell, SARS-CoV-2, neutralizing antibodies

## Abstract

Nanoparticle immunogens excel at rapidly inducing high levels of circulating antibodies and are being deployed as part of several novel vaccines. However, their ability to elicit memory B cell responses is less well understood. Here, we compared serologic and memory B cell responses after prime boost vaccination with either SARS-CoV-2 Wuhan-Hu-1 mRNA vaccine, or protein nanoparticles: SARS-CoV-2 B.1.351 homotypic containing a single receptor binding domain (RBD); (homotypic beta) or a combination of different Sarbecovirus RBDs (mosaic 8b), respectively. The memory B cells elicited by the 3 vaccine regimens showed closely related antibody sequences, similar levels of somatic mutation, and clonal diversity. The breadth of serologic responses elicited by the mosaic nanoparticles was comparable to the homotypic nanoparticle and superior to the mRNA vaccine for some mismatched strains. However, serum neutralizing titers to SARS-CoV-2 were highest after mRNA vaccination. The three vaccines elicited memory B cells that produced antibodies specific to a broad range of epitopes on the RBD that differed in a way that may reflect epitope masking. Monoclonal antibodies derived from memory B cells elicited by the mosaic 8b nanoparticle showed greater breadth against a panel of SARS-CoV-2 variants and SARS-CoV.

## Significance

Nanoparticle vaccines are promising next-generation vaccine candidates, yet their capacity to generate durable memory B cell responses remains incompletely understood. We compared immune responses following SARS-CoV-2 mRNA, homotypic beta nanoparticle, or mosaic 8b nanoparticle vaccination in mice. Serum antibody neutralizing responses against a panel of SARS-CoV-2 variants elicited by an mRNA vaccine were equivalent or superior to those elicited by mosaic 8b nanoparticle vaccines. However, the monoclonal antibodies derived from memory B cells elicited by the mosaic 8b nanoparticle showed better neutralizing breadth against heterologous pseudoviruses. These findings highlight individual strengths of mRNA and nanoparticle vaccines and show that mosaic 8b nanoparticle immunogens can enhance the breadth of memory B cell–derived antibodies.

Vaccine efficacy depends on both cellular and humoral immunity. Whereas circulating antibodies can prevent infection, memory T cells and B cells respond rapidly to breakthrough infection and can limit disease progression. Long-lived circulating antibodies are produced by plasma cells that are selected by a mechanism which favors high-affinity antibody production (1–6). In contrast, memory B cell selection is governed by a distinct mechanism that produces cells with diverse affinity (7–9). As a result, the memory compartment in immunized mice includes B cells producing antibodies that are cross-reactive to antigens that are related but not identical to the immunogen (8–10).

The recent coronavirus pandemic enabled comparative studies of human memory B cell development in response to SARS-CoV-2 infection and/or vaccination (11–15). Several different vaccines were studied and compared to natural infection for their ability to elicit memory B cells (16–20). Overall, the data indicated that mRNA vaccination was most effective in producing memory B cells and preventing serious disease in individuals with breakthrough infections (21, 22).

Humans receiving mRNA vaccines developed a memory B cell compartment that evolved increased breadth and potency over time (15, 23–25). Thus, a significant fraction of memory B cell antibodies elicited by an mRNA vaccine encoding the original Wuhan-Hu-1 Spike also neutralized emerging variants (23, 24, 26). Increasing breadth after SARS-CoV-2 mRNA vaccination was attributed to changes in epitope targeting directed by antibody masking (27). The cross-reactive memory B cells likely contributed to the observation that breakthrough infections with variants of concern were less likely to result in serious disease in vaccinated individuals (28–30).

Coronaviruses are found in many ecological niches and are particularly common in bat species, which make up 20% of all mammals (31). Therefore, future coronavirus spillovers into the human population are likely (32). Memory B cell responses to the current SARS-CoV-2 mRNA vaccines are not universally effective against distantly related coronaviruses making it imperative to explore vaccine strategies that protect against a broader group of these viruses. One of the vaccine concepts that appears to broaden the immune response involves the use of mosaic nanoparticles that display a mixture of different coronavirus receptor binding domains (RBDs), the primary target of neutralizing antibodies (33–35).

Nanoparticle vaccine concepts are also being tested for Influenza virus and HIV-1 (36–40). In all cases, nanoparticle vaccines elicit protective titers of circulating antibodies. However, the impact of nanoparticle vaccines on B cell memory development has not been evaluated in comparison to mRNA vaccination (33, 34, 41–44). Here, we examine the development of serum antibody and B cell memory responses to homotypic beta and mosaic 8b RBD nanoparticle vaccination and compare them to responses elicited by SARS-CoV-2 mRNA vaccines in naïve mice.

## Results

**Fig. 1.**
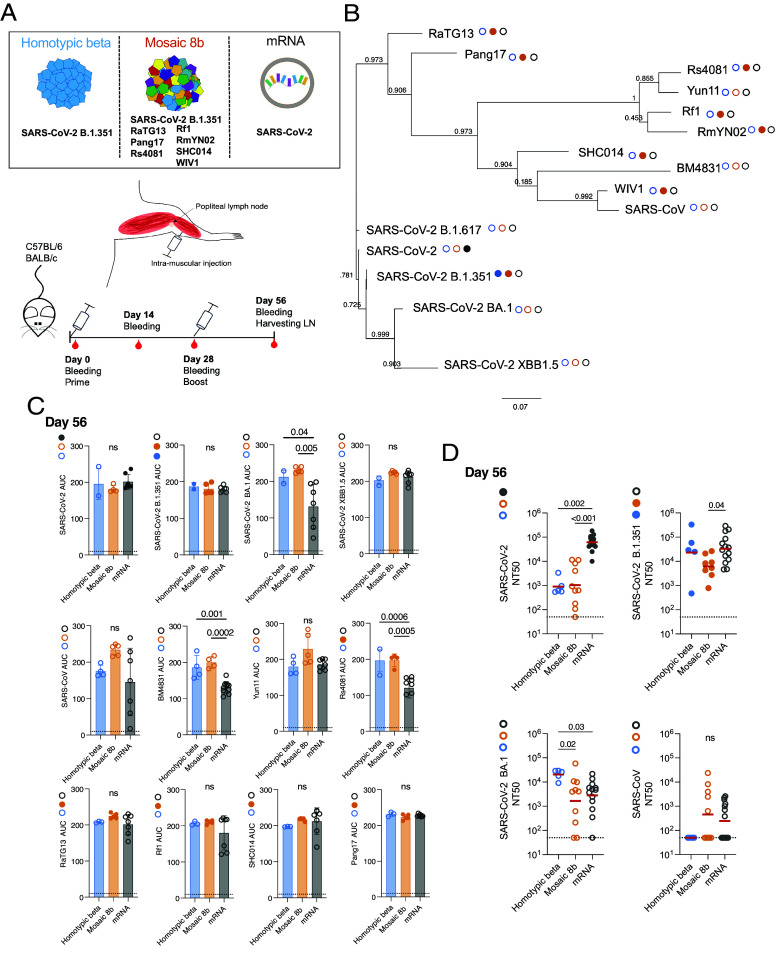
Plasma binding and neutralizing activity. (*A*) Schematic representation of the vaccines and immunization protocol. (*B*) Phylogenetic tree showing amino acid proximity of SARS-CoV-2 RBD with other Sarbecoviruses. Filled circles next to strain names designate strains that are represented in the vaccine (matched) and unfilled circles designate strains that are not represented in the vaccine (mismatched). (*C*) Graphs show plasma IgG binding activity for samples obtained from C57BL/6 mice as normalized area under the curve (AUC) values measured by ELISA for: SARS-CoV-2, SARS-CoV-2 B.1.351, SARS-CoV-2 BA.1, SARS-CoV-2 XBB1.5, SARS-CoV, BM4831, Yun11, Rs4081, Pang17, RaTG13, Rf1, and SHC014 RBDs on day 56. Solid colored dots indicate the presence of the RBD on the immunogen [homotypic beta (blue) mosaic 8b (orange) mRNA (gray)]. Filed circles represent the presence of the RBD in the immunogen. The dotted black line represents negative control. Each dot represents one animal. *P* values were calculated using an ANOVA test. (*D*) Plasma neutralizing titers for SARS-CoV-2, SARS-CoV-2 B.1.351, SARS-CoV-2 BA.1, and SARS-CoV for samples obtained from C57BL/6 and Balb/c mice (homotypic beta: n = 3 and n = 2, mosaic 8b: n = 5 and n = 5, mRNA: n = 7 and n = 7 for C57BL/6 and Balb/c mice, respectively). Plasma NT50s for homotypic (blue), mosaic 8b (orange), and mRNA (gray) measured on day 56. Each dot represents one animal. The horizontal bar represents the geometric mean. All experiments were performed at least twice. *P* values were calculated using the Kruskal–Wallis test followed by Dunn’s multiple comparison test. Only *P* values > 0.05 are shown on the graph.

To examine memory B cell responses to candidate nanoparticle vaccines, we primed C57BL/6J and BALB/c naïve mice with SpyCatcher003-mi3 nanoparticles coupled to SpyTag003-RBDs (33) (*SI Appendix*, Fig. S1 *A* and *B*). The naïve mice were boosted 28 d after the prime with the same formulation (Fig. 1 *A* and *B*). The nanoparticles carried either SARS-CoV-2 B.1.351 RBD (homotypic beta), or a combination of eight different RBDs corresponding to SARS-CoV-2 B.1.351, RaTG13, Pang17, Rf1, Rs4081, SHC014, RmYN02, and WIV1 (mosaic 8b nanoparticles) (Fig. 1 *A* and *B* and *SI Appendix,* Table S1) (33). This collection of RBDs includes sequences representative of sarbecoviruses corresponding to clades 1, 2, 1/2, and 3, covering a broad spectrum of genetic diversity within this subgenus (33). A separate cohort of control naïve mice was primed and boosted 28 d after the prime with the original monovalent SARS-CoV-2 mRNA vaccine encoding a prefusion stabilized Wuhan-Hu-1 spike protein (45) (Fig. 1 *A* and *C*).

### Plasma Binding Activity.

Antibody titers to RBD were measured 14 d after the prime, shortly before the boost on day 28, and 4 wk after the boost on day 56 (Fig. 1*C* and *SI Appendix*, Fig. S1 *A* and *B*). Twelve different RBDs were used to assess binding by ELISA: SARS-CoV-2 which is encoded by the mRNA vaccine; SARS-CoV-2 B.1.351 (46) present on homotypic beta and mosaic 8b particles; Rs4081, Pang17, RaTG13, Rf1, SHC014 found exclusively on the mosaic 8b nanoparticle; and 5 RBDs not found on any of the immunogens: SARS-CoV-2 XBB1.5, SARS-CoV-2 BA.1, BM4831, SARS-CoV, and Yun11 [Fig. 1 *B* and *D* and *SI Appendix,* Fig. S1 *A* and *B* (47–49)].

Two weeks after the prime, there were no significant differences in serum binding titers to 5 of the 12 RBDs tested between the vaccination groups (*SI Appendix,* Fig. S1*A*). The exceptions were SARS-CoV against which the mosaic 8b nanoparticle and mRNA produced higher binding antibodies than homotypic beta and BM4831, Yun11, Rs4081, Pang17, RaTG13, and Rf1, where mosaic 8b was best (*SI Appendix,* Fig. S1*A*). These differences were slightly less apparent 28 d after the prime (*SI Appendix,* Fig. S1*B*).

In all three vaccination groups, boosting produced higher antibody titers (Fig. 1*C*). After the boost there were no significant differences between homotypic beta and mosaic 8b nanoparticle immunization (Fig. 1*C*); however, both were significantly better than the mRNA vaccine against SARS-CoV-2 BA.1, BM4831, and Rs4081 (Fig. 1*C*). Thus, the breadth and overall amount of serologic RBD binding activity was better after nanoparticle than mRNA vaccination in naïve mice.

### Plasma Neutralization Activity.

To measure serum neutralizing activity, we used HIV-1-based virions carrying a Nanoluciferase reporter pseudotyped with either SARS-CoV-2 WT, SARS-CoV-2 B.1.351, SARS-CoV-2 BA.1, or SARS-CoV spikes (50) (Fig. 1*D* and *SI Appendix,* Fig. S1 *C* and *D*). mRNA-vaccinated mice showed significantly higher neutralizing activity against SARS-CoV-2 than either of the nanoparticle vaccines after both prime and boost (Fig. 1*D* and *SI Appendix,* Fig. S1 *C* and *D*). After the boost, mRNA-elicited titers against SARS-CoV-2 B.1.351 were significantly higher than after mosaic 8b vaccination (Fig. 1*D*), and homotypic beta nanoparticle-vaccinated naïve mice showed significantly higher neutralizing titers against SARS-CoV-2 BA.1 than naïve mice vaccinated either with mRNA or mosaic 8b nanoparticles. Furthermore, the amount of SARS-CoV neutralizing activity elicited by the mosaic 8b after boosting was not significantly different from the SARS-CoV-2 mRNA vaccination (Fig. 1*D* and *SI Appendix,* Fig. S1 *C* and *D*).

### Memory B Cells.

**Fig. 2.**
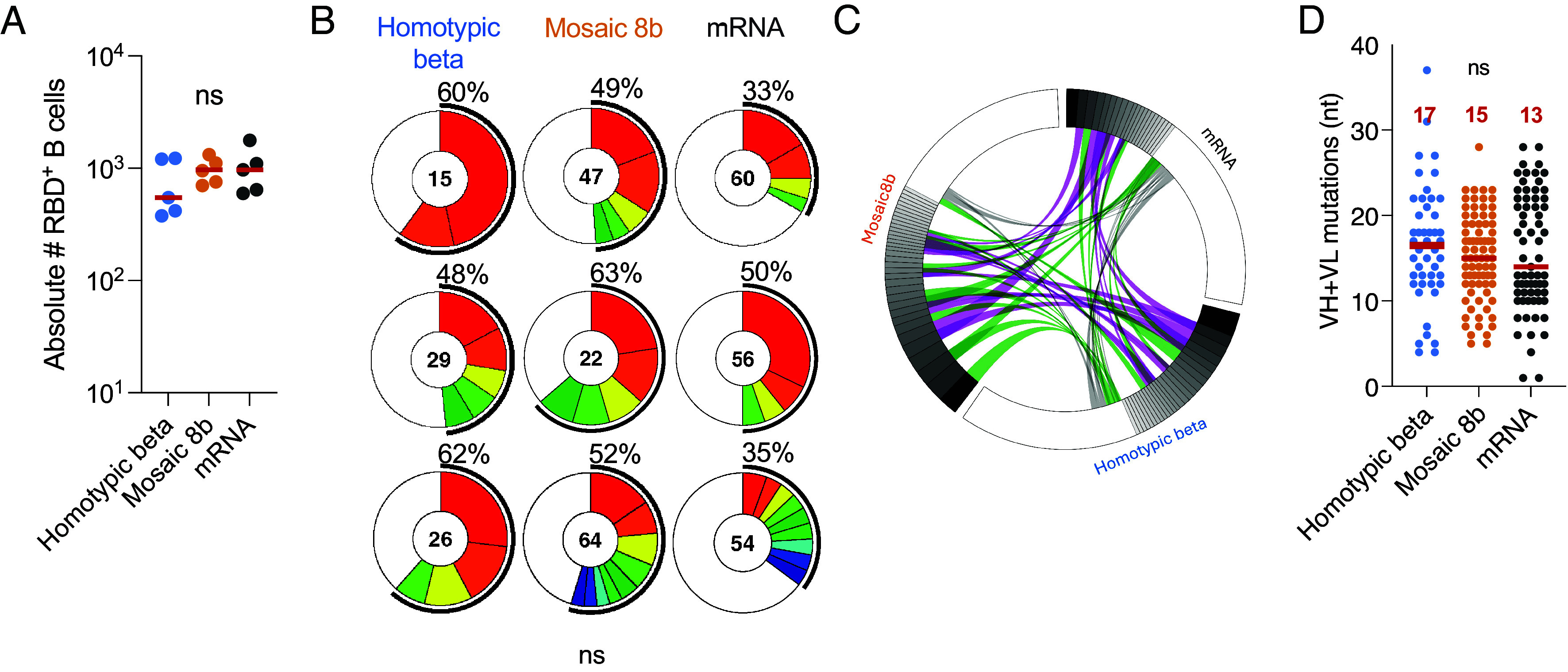
Memory B cell antibodies. (*A*) Absolute number of RBD binding B cells in homotypic beta- (n = 5 in blue), mosaic 8b- (n = 5 in orange), and mRNA- (n = 5 in gray) vaccinated mice obtained from draining lymph node on day 56. (*B*) Pie charts showing the proportional clonal representation of antibodies obtained from the memory B cells in (*A*). The number indicates the number of sequences. The black outline and percentage show the frequency of clonally expanded B cells. Colors represent individual clones. (*C*) The circus plot shows V + J antibody gene segments shared among mice immunized with the different immunogens. Purple shows antibodies shared within clones. Green shows antibodies shared from clones to singles and gray shows shared single antibodies between the 3 immunizations. (*D*) Nucleotide somatic hypermutations (SHM) in the heavy and light chains for all sequences shown in (*B*) for homotypic beta (blue), mosaic 8b (orange), and mRNA (gray) immunization groups. The red line represents the median value. Statistical significance was determined using an ANOVA test.

Memory B cells are responsible for anamnestic serologic responses (51). These cells are selected by different mechanisms than serum antibody-producing plasma cells (52, 53). To examine the antigen-binding memory B cell compartment in the immunized naïve mice, we purified lymph node B cells that bind to SARS-CoV-2 RBD 4 wk after the boost (Day 56) and cloned their antibody genes (Fig. 2 and *SI Appendix*, Table S2 and Fig. S2 *A* and *B*). We selected SARS-CoV-2 for memory B cell capture because this RBD and SARS-CoV-2 B.1.351 had the same serological titers at day 56 and no significant difference in the three groups of vaccines. The absolute number of B cells binding to RBD was similar in the three groups of immunized mice (Fig. 2*A* and *SI Appendix,* Fig. S2*B*). 309 Ig heavy and light chain pairs were obtained, many of which were found in expanded clones (Fig. 2*B* and *SI Appendix,* Table S2). These antibodies from homotypic beta nanoparticle, mosaic 8b nanoparticle, and mRNA vaccination showed closely related V + J sequences (Fig. 2*C* and *SI Appendix,* Table S2). In addition, the antibodies had similar levels of diversity and CDR3 length (*SI Appendix,* Fig. S2 *C* and *D*). Finally, the relative amount of clonal expansion was similar for all 3 immunization regimens, as was the amount of somatic mutation (Fig. 2 *B* and *D*).

### Monoclonal Antibody Binding Activity.

**Fig. 3.**
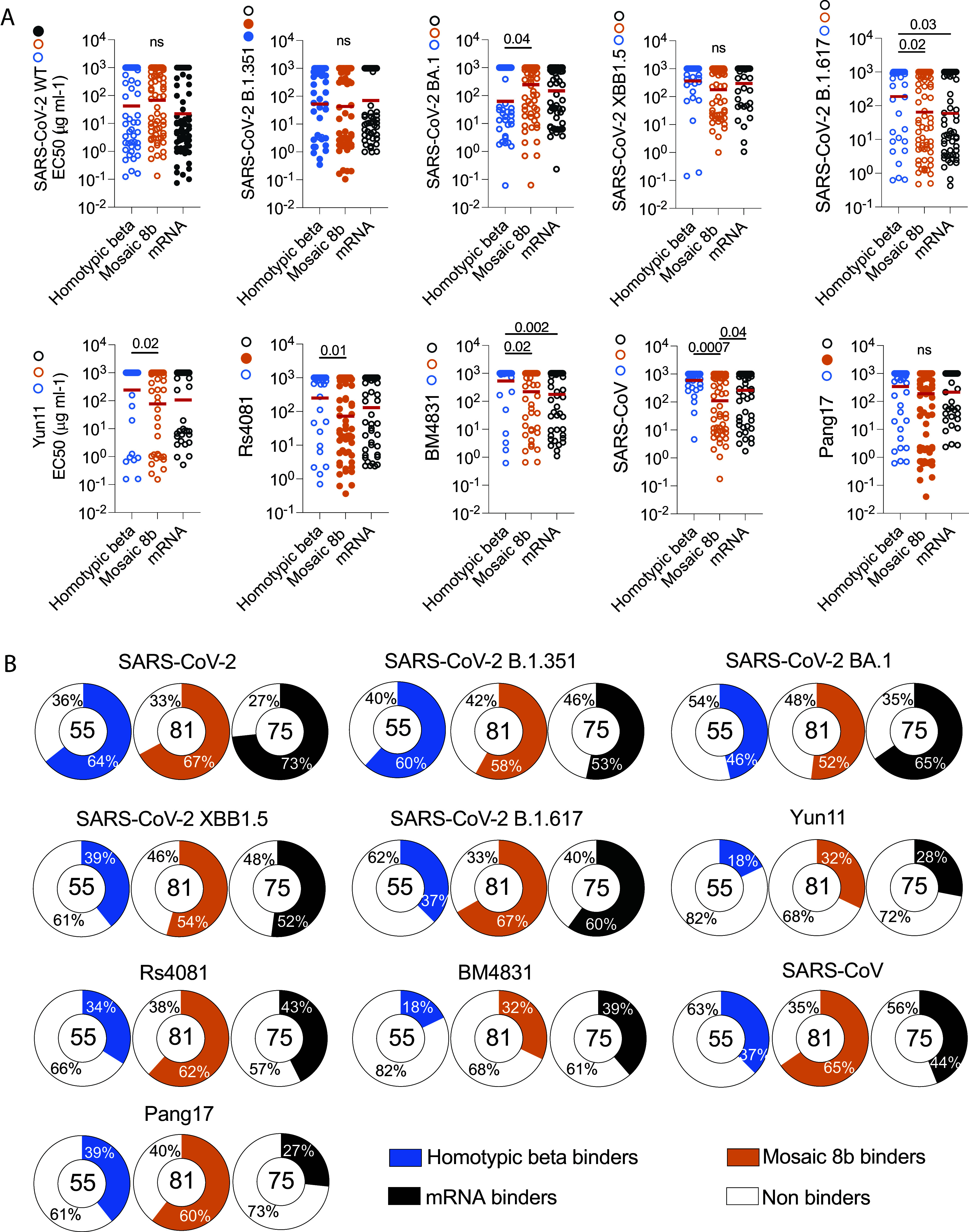
Binding activity of monoclonal antibodies. (*A*) Graphs show monoclonal antibody binding activity measured by ELISA (EC50s) for: SARS-CoV-2, SARS-CoV-2 B.1.351, SARS-CoV-2 BA.1, SARS-CoV-2 XBB1.5, SARS-CoV-2 B.1.617, Yun11, Rs4081, BM4831, SARS-CoV, Pang17 RBDs. Filled colored dots show presence of the respective RBD on the immunogen [homotypic beta (blue) mosaic 8b (orange) mRNA (gray)]. Each dot represents one monoclonal antibody. Red lines indicate geometric mean. Statistical significance was determined using an ANOVA test. Only *P* values > 0.05 are shown on the graph. (*B*) Pie charts indicated the percentage of binding vs. nonbinding antibodies for SARS-CoV-2, SARS-CoV-2 B.1.351, SARS-CoV-2 BA.1, SARS-CoV-2 XBB1.5, SARS-CoV-2 B.1.617, Yun11, Rs4081, BM4831, SARS-CoV, Pang17 RBDs for homotypic beta (blue), mosaic 8b (orange), and mRNA (gray) immunization.

To further characterize the memory response, we expressed 211 randomly selected monoclonal antibodies: 55 from homotypic beta-, 81 from mosaic 8b-, and 75 from mRNA-immunized naïve mice (*SI Appendix,* Table S3). Binding activity was measured by ELISA against SARS-CoV-2 RBD (only present on the mRNA), SARS-CoV-2 B.1.351 RBD (present on homotypic beta and mosaic 8b nanoparticle), Rs4081 and Pang17 RBDs (only present on mosaic 8b), and SARS-CoV-2 BA.1, SARS-CoV-2 XBB1.5, SARS-CoV-2 B.1.617, Yun11, BM4831, and SARS-CoV RBDs (absent from all vaccines; Figs. 1*B* and 3 *A* and *B*). For all RBDs tested, binding by the mosaic8b-elicited antibodies was similar to those elicited by the mRNA vaccine, except for SARS-CoV RBD for which binding by mosaic 8b-elicited antibodies was better (Fig. 3*A*). The mosaic 8b nanoparticle antibodies were significantly better binders than homotypic beta-elicited antibodies to SARS-CoV-2 B.1.617, Yun11, Rs4081, BM4831, and SARS-CoV (Fig. 3*A*). These results are in line with the overall frequency of binders elicited by each vaccine (Fig. 3*B*). For each immunization, a fair percentage of antibodies did not bind to RBDs in ELISAs (Fig. 3*B*). This percentage of nonbinder was similar for each RBDs tested from the three groups of immunization (Fig. 3*B*).

### Monoclonal Antibody Neutralization Activity.

**Fig. 4.**
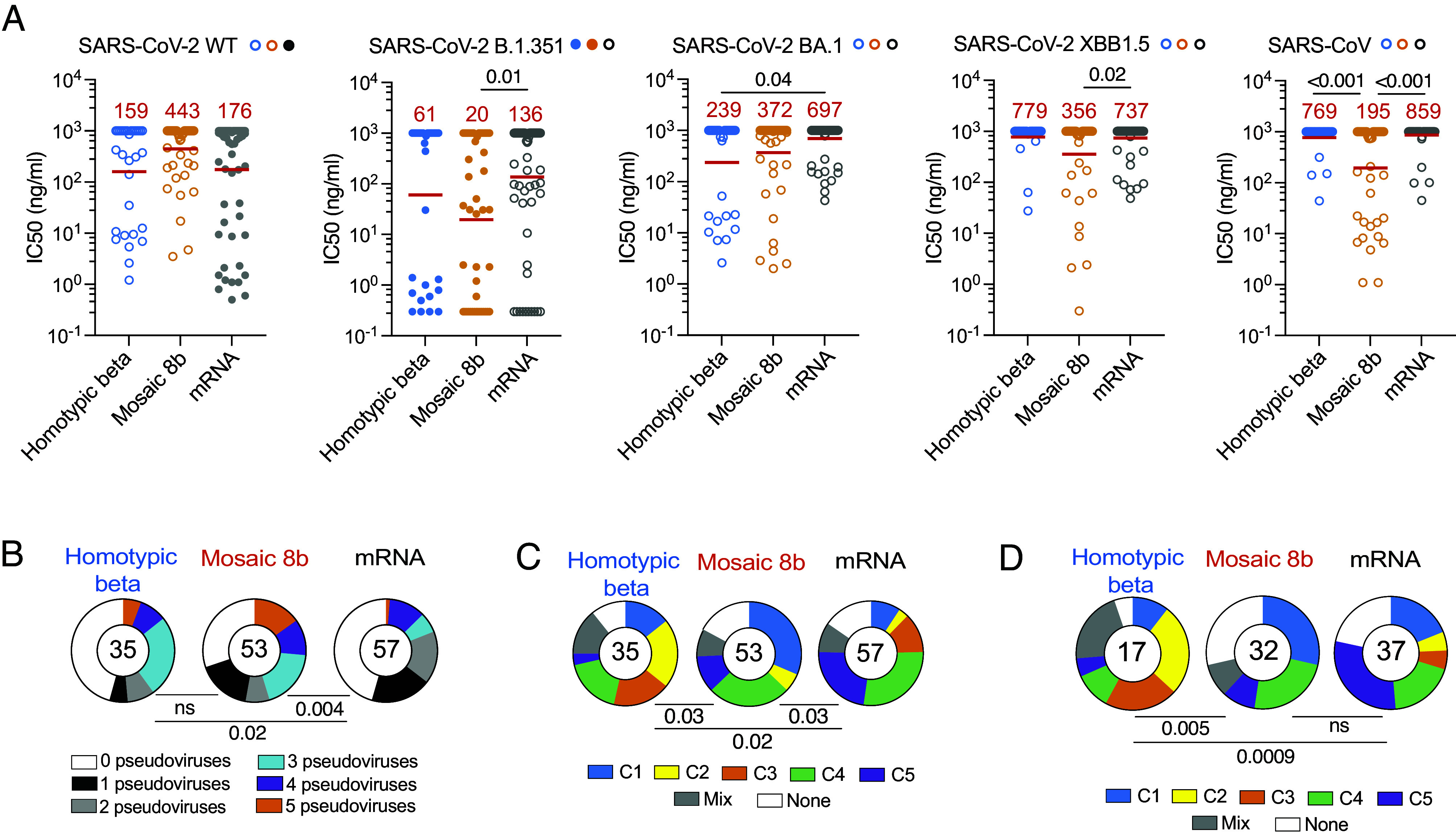
Neutralizing activity and target epitopes of monoclonal antibodies. (*A*) SARS-CoV-2, SARS-CoV-2 B.1.351, SARS-CoV-2 BA.1, SARS-CoV-2 XBB1.5, and SARS-CoV pseudovirus neutralization activity. IC50 values in ng/mL for the indicated pseudoviruses. The red line shows the geometric mean indicated in red. Statistical significance was determined using the Kruskal–Wallis test with subsequent Dunn’s multiple-comparisons test. (*B*) Pie charts depict the fraction of antibodies that neutralize 0 (white), 1 (black), 2 (gray), 3 (blue), 4 (purple), and 5 (orange) pseudoviruses with an IC50 < 1,000 ng/mL. The number indicates the number of antibodies tested. Statistical significance was determined using the two-sided Fisher’s exact tests. (*C*) The pie chart represents epitope target class for all monoclonal antibodies with binding activity to SARS-CoV-2 RBD determined by competition BLI. The number indicates the number of antibodies tested. (*D*) The pie chart represents epitope target class for all monoclonal antibodies that neutralize more than 1 pseudovirus. The number indicates the number of antibodies tested. Statistical significance in *B*–*D* was determined using two-sided Fisher’s exact tests.

To determine the neutralizing activity of the memory antibodies, we tested all 144 antibodies with detectable binding activity to SARS-CoV-2 or SARS-CoV-2 B.1.351 against viruses pseudotyped with SARS-CoV-2, SARS-CoV-2 B.1.351, SARS-CoV-2 BA.1, SARS-CoV-2 XBB1.5, and SARS-CoV spikes (31 from homotypic beta nanoparticle-, 47 from mosaic 8b nanoparticle-, and 66 from mRNA-immunized mice). Overall, 61%, 74%, and 62% of the binding antibodies cloned from memory B cells obtained from homotypic beta, mosaic 8b nanoparticle, and mRNA vaccinated naïve mice, respectively, showed measurable neutralizing activity to SARS-CoV-2 or SARS-CoV-2 B.1.351 with an IC50 of less than 1 µg/mL (Fig. 4*A* and *SI Appendix,* Fig. S3*A*).

The fraction of SARS-CoV-2 neutralizers obtained from mRNA immunized mice is consistent with the fraction obtained from vaccinated humans (19). The geometric mean of the neutralizing activity was 159, 443, and 176 ng/mL for the homotypic beta, mosaic 8b nanoparticles, and

mRNA memory antibodies, respectively, with no significant difference between the mosaic8b-elicited antibodies and either homotypic beta- or mRNA vaccine-elicited antibodies (Fig. 4*A* and *SI Appendix,* Fig. S3*A*). Although the number of antibodies is limited, these trends remain the same when considering neutralizing antibodies alone (*SI Appendix,* Fig. S3*B*). Mosaic 8b-derived antibodies had significantly better neutralizing activity against SARS-CoV-2 B.1.351 and SARS-CoV-2 XBB1.5 than the mRNA vaccine-derived antibodies (Fig. 4*A* and *SI Appendix,* Fig. S3*A*). As might be expected, antibodies derived from mice vaccinated with the mosaic 8b nanoparticle were significantly better neutralizers of SARS-CoV than the other two immunogens (Fig. 4*A* and *SI Appendix,* Fig. S3*A*). However, there were no statistically significant differences between the memory antibodies elicited by the mosaic 8b nanoparticles vs. the other two for neutralization of SARS-CoV-2 BA.1-pseudotyped viruses (Fig. 4*A* and *SI Appendix,* Fig. S3*A*).

The breadth of neutralizing activity was evaluated by comparing the ability of any one antibody to neutralize 0 to 5 of the pseudotyped viruses (Fig. 4*B* and *SI Appendix,* Fig. S3*A*). The distribution of antibodies neutralizing 0 to 5 strains elicited by homotypic beta and mosaic 8b nanoparticles was not significantly different (Fig. 4*B* and *SI Appendix,* Fig. S3*A*). Moreover, both sets of nanoparticle vaccine antibodies showed increased overall breadth of neutralization when compared to the mRNA vaccine (Fig. 4*B* and *SI Appendix,* Fig. S3*A*).

### Epitopes Targeted by Monoclonal Antibodies.

Memory antibodies elicited after the initial mRNA prime and boost in humans typically target Class 1 and 2 epitopes that overlap with the ACE2 binding site of the RBD (51, 54). After the 3rd dose, antibody-mediated epitope masking shifts the immune response to target more conserved features of the RBD between Class 3, 4, and 5 domains (23). To determine the specificity of the memory antibodies elicited by nanoparticle and mRNA prime-boost vaccination in mice, we performed biolayer interferometry (BLI) experiments in which a preformed antibody-RBD immune complex was exposed to a second monoclonal (reference) with predetermined specificity (55). The 5 reference antibodies, C022, C144, C135, C837, and C5078, bind to Class 1, 2, 3, 4, 5, respectively [Fig. 4*C* and *SI Appendix,* Fig. S4 (56)]. Antibodies that bound to Class 1 or 2 (blue and yellow) were more prominent in the memory antibodies obtained from homotypic beta and mosaic 8b nanoparticle- than mRNA- immunized naive mice (Fig. 4*C* and *SI Appendix,* Fig. S4). In contrast, the memory antibodies elicited by mRNA immunization were significantly different from the nanoparticle antibodies in that they primarily targeted the Class 3, 4, and 5 domains of the RBD (Fig. 4*C* and *SI Appendix,* Fig. S4).

Broader antibodies with neutralizing activity against more than 1 pseudovirus also differed significantly between the groups. The combined class 3/4/5 antibodies that target the more conserved region of the RBD were more highly represented among neutralizers elicited by the mRNA vaccine than among those obtained from homotypic beta and mosaic 8b vaccination (Fig. 4*D*).

In conclusion, the mRNA vaccine elicits memory B cells producing antibodies that are less broad than those elicited by homotypic beta or mosaic 8b nanoparticle vaccines.

## Discussion

We compared serologic and memory B cell responses elicited by two different nanoparticle vaccines to those elicited by an mRNA vaccine in naïve mice. An adjuvant was added to the three vaccines to attain consistent T cell activation. Consistent with prior analysis (33), the binding activity of antibodies in serum elicited by the mosaic 8b nanoparticle vaccine carrying a diverse collection of coronavirus RBDs was somewhat broader than those elicited by an mRNA vaccine. When comparing serum neutralizing activity, the mRNA-vaccine elicited significantly higher neutralizing titers to its cognate antigen, SARS-CoV-2, and titers equivalent to the nanoparticles against their cognate antigen, SARS-CoV-2 B.1.351.

Nanoparticles can resemble pathogens in that they display multiple copies of antigen on their surface (57). These repeating units can cross-link antigen-specific receptors on B lymphocytes and produce rapid antibody responses that help clear the pathogen (58, 59). Consistent with this idea, mosaic 8b nanoparticle vaccination elicited a rapid initial antibody response that was protective against infection with either SARS-CoV-2 or SARS-CoV in experimental animals (33, 34, 41, 43, 44).

Memory B cell responses produced by nanoparticle vaccination showed similar levels of somatic mutation to those elicited by mRNA vaccination, suggesting that memory cells that develop after nanoparticle and mRNA vaccination do so after equivalent numbers of cycles of mutation and selection in germinal centers (60). However, the relative fraction of B cells producing anti-RBD neutralizing antibodies was slightly higher for animals receiving the mRNA vaccine. This may in part be due to the mRNA vaccine resulting in expression of a complete stabilized spike in its native conformation, whereas the RBD displayed on the nanoparticle is devoid of other components of the spike that may block non-neutralizing epitopes on the RBD. In addition, the relative effects of dosing cannot be excluded, and how these results obtained in mice might translate to humans is difficult to predict. Furthermore, our results model primary immunity in naïve mice, whereas in clinical practice, the majority of vaccines would be used in the setting of existing B cell immunity from prior vaccination or infection.

Germinal center responses are not required for memory B cell formation, but memory cells emerging from germinal centers are more likely to carry somatic mutations (60, 61). The mutations diversify the germinal center-derived memory B cells resulting in a collection of cells some of which produce highly specific antibodies and others that may also be able to recognize related antigens (8–10). Thus, germinal center diversification and selection produce memory B cells that can be called upon to respond to and neutralize related antigenic variants (11, 12, 23, 24). In addition to T cell responses to conserved epitopes, the diversified memory B cell compartment elicited by mRNA vaccination likely contributed to protection from the serious consequences of infection in individuals who experienced breakthrough infections with SARS-CoV-2 variants after mRNA vaccination (28–30).

Although all 3 vaccines appeared to elicit comparable germinal center responses, the epitopes targeted by memory B cells differed between nanoparticle and mRNA vaccines. In both humans and mice, the initial response to mRNA vaccination is dominated by antibodies targeting the more exposed Class 1 and 2 epitopes with a shift to less accessible Class 3,4,5 epitopes after a boost due to epitope masking (27). Compared to this, the fraction of Class 3,4,5 antibodies is reduced after a nanoparticle boost. In addition to the topological differences in the antigen presented by the immunogens, the higher overall level of Class 3,4,5 antibodies that develop after mRNA immunization may be due to initial epitope masking by Class 1 and 2 antibodies (27).

In summary, both SARS-CoV-2 mRNA and nanoparticle vaccines elicit germinal center reactions that promote production of a diverse memory B cell compartment enriched in neutralizing antibodies (15, 18, 23, 62). Mosaic 8b nanoparticles produce somewhat higher levels of memory antibodies with increased breadth and potency against heterologous viruses. However, SARS-CoV-2 mRNA vaccination elicits comparable serologic neutralizing responses after prime boost vaccination.

## Materials and Methods

### RBD Expression and Preparation of RBD-mi3 Nanoparticles.

RBD expression and preparation of SpyCatcher003-mi3 particles was done as previously described (19, 44).

### Phylogenetic Tree.

A sequence alignment of Sarbecovirus RBDs was built using Geneious software. A phylogenetic tree was calculated from the amino acid alignment using Geneious software. A table with the percentage of amino acid identity was also generated (*SI Appendix,* Table S1). All the RBD sequences can be found in GenBank; RaTG13-CoV (GenBank QHR63300; S protein residues 319–541), SHC014-CoV (GenBank KC881005; residues 307–524), Rs4081-CoV (GenBank KY417143; S protein residues 310–515), pangolin17-CoV (GenBank QIA48632; residues 317–539), RmYN02-CoV (GSAID EPI_ISL_412977; residues 298–503), Rf1-CoV (GenBank DQ412042; residues 310–515), WIV1-CoV (GenBank KF367457; residues 307–528), Yun11-CoV (GenBank JX993988; residues 310–515), and BM4831-CoV (GenBank NC014470; residues 310–530).

### Mouse Immunization.

Female mice (C57BL/6 and BALB/c) purchased from Jackson aged 6 to 12 wk were housed at a temperature of 22 °C and a humidity of 30 to 70% under a 12 h to 12 h light–dark cycle with ad libitum access to food and water. Mice were primed with homotypic beta (SARS-CoV-2 B1.351 RBD) nanoparticles (n = 5), mosaic 8b nanoparticles (n = 10), or Spikevax Moderna mRNA vaccine (n = 10). Mice were bled on days 14 and 28 after immunization and boosted after the second bleed. Lymph nodes were harvested on day 56 after immunization. Homotypic beta and mosaic 8b nanoparticles were injected intramuscularly. Intramuscular injections were performed using 1 μg of mRNA vaccine (1 μg of mRNA vaccine in 50 μL of PBS + 50 μL Addavax) or 5 μg of nanoparticles in 50 μL of PBS + 50 μL of Addavax (Invivogen). The experiments were performed according to protocols approved by the Rockefeller University Institutional Animal Care and Use Committee (IACUC).

### Mouse Sampling.

Serum was collected with Goldenrod animal lancet by submandibular puncture at intermediary time points and cardiac puncture when the experiments were terminated in Serum gel Z1/1.1 (SARSTEDT), centrifuged at 1,500 rpm for 1 min and stored at −20 °C. Lymph nodes were dissected, mashed through a 70-μm cell strainer with 5 mL of RPMI and the cells labeled for flow cytometry or frozen in fetal bovine serum (FBS) with 10% dimethyl sulfoxide in a gradual-freezing (~1 °C/min) container. Frozen cells were thawed in a 37 °C water bath and immediately transferred to a prewarmed medium consisting of RPMI 1640, supplemented with 10% heat-inactivated FBS, 10 mM HEPES, 1× antibiotic–antimycotic, 1 mM sodium pyruvate, 2 mM L-glutamine, and 53 μM 2-mercaptoethanol (all from Gibco).

### ELISAs.

We performed enzyme-linked immunosorbent assays (ELISAs) to evaluate plasma and monoclonal antibodies binding capacity to a panel of RBDs from SARS-CoV-2 (Wuhan-Hu-1), SARS-CoV-2 Omicron BA.1, SARS-CoV-2 Omicron XBB1.5, SARS-CoV-2 Delta (B.1.617), SARS-CoV-2 B1.351, Rs4081, BM4831, Yun11, SARS-CoV, Pang17, RatG13, Rf1, SHC014. 50 μL of a 1 μg/mL solution of the relevant RBD in Phosphate-buffered Saline (PBS) was coated on high-binding 96-half-well plates (Corning 3690) overnight at 4 °C. Plates were washed six times with washing buffer [1× PBS with 0.05% Tween-20 (Sigma-Aldrich)] and further incubated for 2 h at room temperature with 170 μL of blocking buffer [1× PBS with 1% BSA and 0.05% Tween-20 (Sigma) and 0.1 mM EDTA]. Following the blocking step, plasma samples or monoclonal antibodies were added in PBS and incubated for 1 h at room temperature. Plasma samples were diluted (1:50) and serially diluted (1:3) 10 times. Monoclonal antibodies were used at 10 μg/mL starting concentration and serially diluted (1:4) 10 times. Plates were washed six times with washing buffer and incubated with secondary antibody (anti-human IgG for monoclonal and Anti-Mouse IgG for Plasma samples) conjugated to horseradish peroxidase (HRP) (Jackson Immuno Research 109-036-088 109-035-129 and Sigma A0295) in blocking buffer (1:5,000). Finally, plates were washed six times with washing buffer and further developed by addition of 50 μL of the HRP substrate, 3,3′,5,5′-Tetramethylbenzidine (TMB) (ThermoFisher) for 5 min. The reaction was stopped with 50 μL of 1 M H_2_SO_4_ solution and absorbance was measured at 450 nm with ELISA microplate reader (FluoStar Omega, BMG Labtech). Omega and Omega MARS software were used for the data analysis. A positive control monoclonal antibody C837 was used for anti-RBD ELISA and added to every plate for normalization for plasma samples. The average of its signal was used for normalization of all the other values on the same plate with Excel software. We determined the half-maximal binding titer or AUC for plasma using four-parameter nonlinear regression in GraphPad Prism V9.1. 3BNC117, an HIV antibody, was used as a negative control for validation (55). For monoclonal antibodies, half-maximal concentration (EC50) was determined using four-parameter nonlinear regression in GraphPad Prism V9.1. The monoclonal antibodies with an EC50s above 1,000 ng/mL were considered nonbinders. All reported values represent the average of at least three independent experiments.

### SARS-CoV-2 Pseudotyped Reporter Virus.

The plasmids pSARS-CoV-SΔ19 and pSARS-CoV-2-SΔ19 (based on Wuhan-Hu-1 spike) expressing a C-terminally truncated SARS-CoV or SARS-CoV-2 spike protein, respectively, have been described (55, 63). Variant pseudoviruses resembling SARS-CoV-2 variants SARS-CoV-2 B.1.351 (11) and Omicron BA.1 have been described (64), as was a plasmid encoding for SARS-CoV-2 variant Omicron XBB1.5 (56). Plasmids were generated by introduction of substitutions using synthetic gene fragments (IDT) or overlap extension PCR-mediated mutagenesis and Gibson assembly. Specifically, the variant-specific deletions and substitutions introduced were as follows:

Beta B.1.351: D80A, D215G, L242H, R246I, K417N, E484K, N501Y, D614G, and A701V

Omicron BA.1: A67V, Δ69-70, T95I, G142D, Δ143-145, Δ211, L212I, ins214EPE, G339D, S371L, S373P, S375F, K417N, N440K, G446S, S477N, T478K, E484A, Q493K, G496S, Q498R, N501Y, Y505H, T547K, D614G, H655Y, H679K, P681H, N764K, D796Y, N856K, Q954H, N969H, N969K, and L981F

Omicron XBB.1.5: T19I, L24S, del25-27, V83A, G142D, del144, H146Q, Q183E, V213E, G252V, G339H, R346T, L368I, S371F, S373P, S375F, T376A, D405N, R408S, K417N, N440K, V445P, G446S, N460K, S477N, T478K, E484A, F486P, F490S, Q498R, N501Y, Y505H, D614G, H655Y, N679K, P681H, N764K, D796Y, Q954H, and N969K

Deletions/substitutions corresponding to the variants listed above were incorporated into a spike protein that also includes the R683G substitution, which disrupts the furin cleavage site and increases particle infectivity. Neutralizing activity against mutant pseudoviruses were compared to a wildtype (WT) SARS-CoV-2 spike sequence (NC_045512), carrying R683G.

SARS-CoV-2 pseudotyped particles were generated as previously described (55, 63). Briefly, 293T (CRL-11268) cells were obtained from ATCC, and the cells were transfected with pNL4-3ΔEnv-nanoluc and pSARS-CoV-2-SΔ19, particles were harvested 48 h posttransfection, filtered, and stored at −80 °C.

### Pseudotyped Virus Neutralization Assay.

Mouse sera or monoclonal antibodies were fivefold serially diluted and incubated with SARS-CoV-2, SARS-CoV-2 B.1.351, SARS-CoV-2 BA.1, SARS-CoV-2 XBB1.5, or SARS-CoV pseudotyped virus for 1 h at 37 °C. The mixture was subsequently incubated with HT1080/Ace2 cl14 cells for 48 h after which cells were washed with PBS and lysed with Luciferase Cell Culture Lysis 5× reagent (Promega). Nanoluc Luciferase activity in lysates was measured using the Nano-Glo Luciferase Assay System (Promega) with the ClarioStar Microplate Multimode Reader (BMG). The relative luminescence units were normalized to those derived from cells infected with SARS-CoV-2, SARS-CoV-2 B.1.351, SARS-CoV-2 BA.1, SARS-CoV-2 XBB1.5, or SARS-CoV pseudotyped virus in the absence of serum or monoclonal antibodies. The half-maximal neutralization titers for serum (NT50) or half-maximal inhibitory concentrations for monoclonal antibodies (IC50) were determined using four-parameter nonlinear regression (least squares regression method without weighting; constraints: top = 1, bottom = 0) (GraphPad Prism9).

### Biotinylated Protein for Flow Cytometry.

SARS-CoV-2 RBD (Wuhan Hu-1) was produced as an Avi-tagged protein and biotinylated with the Biotin-Protein Ligase-BIRA kit following the manufacturer’s protocol (55). Ovalbumin (Sigma, A5503-1G) was biotinylated using the EZ-Link Sulfo-NHS-LC-Biotinylation kit according to the manufacturer’s instructions (Thermo Scientific). Biotinylated ovalbumin was conjugated to streptavidin-BV421 and used to remove unspecific binding for the single-cell sorting experiments (Biolegend, 40-52-07). SARS-CoV-2 RBD was conjugated to streptavidin-PE (BD Biosciences, 55-40-61) and streptavidin-APC (Biolegend 40-52-07) for single-cell sorting.

### Flow Cytometry and Single-Cell Sorting.

B cells were enriched by negative selection using a pan-B cell isolation kit following the manufacturer’s instructions (Miltenyi Biotec, 130-101-638). Enriched B cell suspension was incubated in fluorescence-activated cell sorting (FACS) buffer [1× PBS, 2% FCS, and 1 mM ethylenediaminetetraacetic acid (EDTA)], washed, and resuspended in a solution of mouse Fc-receptor blocking (BD Pharmigen 1279893), fluorophore-conjugated bait (SARS-CoV-2 RBD-APC+, SARS-CoV-2 RBD-PE+), and Zombie-NIR Live/Dead cell marker for 15 min on ice. A master mix of anti-mouse antibodies (1:200) was used to label the cells for another 30 min incubation; anti-GL7-FITC (BD Pharmigen, 55366), anti-CD38 AF700 (Invitrogen, 56038182) anti-CD95-PECy7 (BD Biosciences, 557653), anti-CD20-BV711 (BD horizon, 563832), anti-CD4-APC-eFluro 780 (Invitrogen, 47004282), anti-CD8-APC-eFluor 780 (Invitrogen, 47008182), anti-NKK1.1-APC-eFluor 780 (Invitrogen, 47594182), and anti-F4/80 APC-eFluro 780 (Invitrogen, 47480182). Single B cell were sorted (CD4−, CD8−, NKK1.1−, GR1−, CD20+, CD95+, GL7+, Ova—RBD-PE+ and RBD-APC+) into individual wells of 96-well plates containing 4 μL of TE buffer + 2% β-Mercaptoethanol using FACS Aria III. We used FACSDiva software (Becton Dickinson) for the acquisition of data and FlowJo software for the analysis. Sorted B cells were frozen on dry ice and stored at −80 °C.

### Antibody Sequencing, Cloning, and Expression.

All monoclonal antibodies were identified and sequenced as described previously (55, 65). Following single-cell sorting, RNA was cleaned up with RNAClean XP (Beckman Coulter, A63987) following the manufacturer’s instructions. Clean RNA from single cells was reverse transcribed with SuperScript III Reverse Transcriptase (Invitrogen, 18080-044). The cDNA was stored at −20 °C or used for nested PCR and Sanger sequencing of the variable IGH and IGK genes. Amplicons from the second PCR (heavy and light chain) were sent for sequencing at Genewiz and further used as templates for sequence- and ligation-independent cloning into human antibody expression vectors. Recombinant monoclonal antibodies were produced and purified as described (55). All the sequence of monoclonal antibodies can be found online at https://doi.org/10.5281/zenodo.17515419.

### Biolayer Interferometry.

We performed BLI experiments to measure antibody affinity with Octet Red (ForteBio) at 30 °C with shaking at 1,000 r.p.m. Epitope binding assays were performed with protein A biosensor (ForteBio 18-5010), following the manufacturer’s protocol: 1) sensor check: sensors immersed 30 s in buffer alone (buffer ForteBio 18-1105), 2) capture 1st Ab: sensors immersed 10 min with Ab1 at 10 μg/mL, 3) baseline: sensors immersed 30 s in buffer alone, 4) blocking: sensors immersed 5 min with IgG isotype control at 10 μg/mL, 5) baseline: sensors immersed 30 s in buffer alone, 6) antigen association: sensors immersed 5 min with RBDs at 10 μg/mL, 7) baseline: sensors immersed 30 s in buffer alone, and 8) association Ab2: sensors immersed 5 min with Ab2 at 10 μg/mL. Curve fitting was performed using Fortebio Octet Data analysis software (ForteBio). Anti-RBD affinity measurements were corrected by subtracting the signal from IgGs in the absence of RBDs. The kinetic analysis using protein A biosensor was performed as follows: 1) baseline: 60 s immersion in buffer, 2) loading: 200 s immersion in a solution with IgGs 10 μg/mL, 3) baseline: 200 s immersion in buffer, 4) association: 300 s immersion in solution with WT SARS-CoV-2 RBD at 20, 10, or 5 μg/mL, and 5) dissociation: 600 s immersion in buffer. Curve fitting was performed using a fast 1:1 binding model and Data analysis software (ForteBio). Mean KD values were determined by averaging all binding curves that matched the theoretical fit with an R^2^ value ≥ 0.8. All reported values represent the average of at least two independent experiments.

### Computational Analyses of Antibody Sequences.

Antibody sequences were trimmed based on quality and annotated using Igblastn v.1.14. with the IMGT domain delineation system. Annotation was performed systematically using Change-O toolkit v.0.4.540 (66). Clonality of heavy and light chains was determined using DefineClones.py implemented by Change-O v0.4.5 (66). The script calculates the Hamming distance between each sequence in the dataset and its nearest neighbor. Distances are subsequently normalized and to account for differences in junction sequence length, and clonality is determined based on a cut-off threshold of 0.15. Heavy and light chains derived from the same cell were subsequently paired, and clonotypes were assigned based on their V and J genes using in-house R and Perl scripts. All scripts and the data used to process antibody sequences are publicly available on GitHub (https://github.com/stratust/igpipeline/tree/igpipeline2_timepoint_v2).

The frequency distributions of mouse V genes in anti-SARS-CoV-2 antibodies from this study were compared to 131,284,220 IgH and IgL sequences generated by Soto et al. (67) and downloaded from cAb-Rep (68), a database of human shared BCR clonotypes available at https://cab-rep.c2b2.columbia.edu/. We selected the IgH and IgL sequences from the database that are partially coded by the same V genes and counted them according to the constant region. The frequencies shown in Fig. S2 are relative to the source and isotype analyzed. We used the two-sided binomial test to check whether the number of sequences belonging to a specific IGHV or IGLV gene in the repertoire is different according to the frequency of the same IgV gene in the database. Adjusted *P*-values were calculated using the false discovery rate (FDR) correction. Significant differences are displayed with numeric values on the graph.

Nucleotide SHM and complementarity-determining region (CDR3) length were determined using in-house R and Perl scripts. For SHM, IGHV and IGLV nucleotide sequences were aligned against their closest germlines using Igblastn, and the number of differences was considered to correspond to nucleotide mutations. The average number of mutations for V genes was calculated by dividing the sum of all nucleotide mutations across all participants by the number of sequences used for the analysis.

## Supplementary Material

Appendix 01 (PDF)

## Data Availability

Figures and statistics were made with GraphPad Prism9 and arranged in Adobe Illustrator 2022. Comparisons between groups (homotypic beta, mosaic 8b, or mRNA) were calculated using an unpaired or paired *t* test in Prism 9.0 or multiple comparison (ANOVA). Differences were considered significant when *P* values were less than 0.05. Computer code to process the antibody sequences is available in ref. 11. All other data are included in the manuscript and/or *SI Appendix*.

## References

[1] T. G. Phan , High affinity germinal center B cells are actively selected into the plasma cell compartment. J. Exp. Med. **203**, 2419–2424 (2006). 17030950 10.1084/jem.20061254 PMC2118125

[2] A. Radbruch , Competence and competition: The challenge of becoming a long-lived plasma cell. Nat. Rev. Immunol. **6**, 741–750 (2006). 16977339 10.1038/nri1886

[3] M. J. Robinson, R. H. Webster, D. M. Tarlinton, How intrinsic and extrinsic regulators of plasma cell survival might intersect for durable humoral immunity. Immunol. Rev. **296**, 87–103 (2020). 32592168 10.1111/imr.12895

[4] K. G. Smith, A. Light, G. J. Nossal, D. M. Tarlinton, The extent of affinity maturation differs between the memory and antibody-forming cell compartments in the primary immune response. EMBO J. **16**, 2996–3006 (1997). 9214617 10.1093/emboj/16.11.2996 PMC1169918

[5] N. J. Krautler , Differentiation of germinal center B cells into plasma cells is initiated by high-affinity antigen and completed by Tfh cells. J. Exp. Med. **214**, 1259–1267 (2017). 28363897 10.1084/jem.20161533 PMC5413338

[6] A. J. MacLean , Affinity maturation of antibody responses is mediated by differential plasma cell proliferation. Science **387**, 413–420 (2025). 39700316 10.1126/science.adr6896 PMC11938350

[7] G. V. Zuccarino-Catania , CD80 and PD-L2 define functionally distinct memory B cell subsets that are independent of antibody isotype. Nat. Immunol. **15**, 631–637 (2014). 24880458 10.1038/ni.2914 PMC4105703

[8] C. Viant , Antibody affinity shapes the choice between memory and germinal center B cell fates. Cell **183**, 1298–1311.e1211 (2020). 33125897 10.1016/j.cell.2020.09.063 PMC7722471

[9] T. Inoue, T. Kurosaki, Memory B cells. Nat. Rev. Immunol. **24**, 5–17 (2023). 37400644 10.1038/s41577-023-00897-3

[10] R. Shinnakasu , Regulated selection of germinal-center cells into the memory B cell compartment. Nat. Immunol. **17**, 861–869 (2016). 27158841 10.1038/ni.3460

[11] Z. Wang , Naturally enhanced neutralizing breadth against SARS-CoV-2 one year after infection. Nature **595**, 426–431 (2021). 34126625 10.1038/s41586-021-03696-9 PMC8277577

[12] Z. Wang , Analysis of memory B cells identifies conserved neutralizing epitopes on the N-terminal domain of variant SARS-Cov-2 spike proteins. Immunity **55**, 998–1012.e1018 (2022). 35447092 10.1016/j.immuni.2022.04.003 PMC8986478

[13] C. Gaebler , Evolution of antibody immunity to SARS-CoV-2. Nature **591**, 639–644 (2021). 33461210 10.1038/s41586-021-03207-w PMC8221082

[14] A. Sokal , Maturation and persistence of the anti-SARS-CoV-2 memory B cell response. Cell **184**, 1201–1213.e1214 (2021). 33571429 10.1016/j.cell.2021.01.050 PMC7994111

[15] W. Kim , Germinal centre-driven maturation of B cell response to mRNA vaccination. Nature **604**, 141–145 (2022). 35168246 10.1038/s41586-022-04527-1 PMC9204750

[16] Z. Wang , Humoral immunity to SARS-CoV-2 elicited by combination COVID-19 vaccination regimens. J. Exp. Med. **219**, e20220826 (2022). 36006380 10.1084/jem.20220826 PMC9418484

[17] A. Cho , Antibody evolution to SARS-CoV-2 after single-dose Ad26.COV2.S vaccine in humans. J. Exp. Med. **219**, e20220732 (2022). 35776090 10.1084/jem.20220732 PMC9253517

[18] A. Cho , Anti-SARS-CoV-2 receptor-binding domain antibody evolution after mRNA vaccination. Nature **600**, 517–522 (2021). 34619745 10.1038/s41586-021-04060-7 PMC8674133

[19] Z. Wang , mRNA vaccine-elicited antibodies to SARS-CoV-2 and circulating variants. Nature **592**, 616–622 (2021). 33567448 10.1038/s41586-021-03324-6 PMC8503938

[20] C. I. Kaku , Broad anti-SARS-CoV-2 antibody immunity induced by heterologous ChAdOx1/mRNA-1273 vaccination. Science **375**, 1041–1047 (2022). 35143256 10.1126/science.abn2688 PMC8939765

[21] W. H. Self , Comparative effectiveness of Moderna, Pfizer-BioNTech, and Janssen (Johnson & Johnson) vaccines in preventing COVID-19 hospitalizations among adults without immunocompromising conditions—United States, March–August 2021. Morb. Mortal. Wkly. Rep. **70**, 1337–1343 (2021). 10.15585/mmwr.mm7038e1 PMC8459899 34555004

[22] M. E. McMenamin , Vaccine effectiveness of one, two, and three doses of BNT162b2 and CoronaVac against COVID-19 in Hong Kong: A population-based observational study. Lancet Infect. Dis. **22**, 1435–1443 (2022). 35850128 10.1016/S1473-3099(22)00345-0 PMC9286709

[23] F. Muecksch , Increased memory B cell potency and breadth after a SARS-CoV-2 mRNA boost. Nature **607**, 128–134 (2022). 35447027 10.1038/s41586-022-04778-y PMC9259484

[24] R. R. Goel , Efficient recall of Omicron-reactive B cell memory after a third dose of SARS-CoV-2 mRNA vaccine. Cell **185**, 1875–1887.e1878 (2022). 35523182 10.1016/j.cell.2022.04.009 PMC8989683

[25] W. B. Alsoussi , SARS-CoV-2 omicron boosting induces de novo B cell response in humans. Nature **617**, 592–598 (2023). 37011668 10.1038/s41586-023-06025-4

[26] R. R. Goel , mRNA vaccines induce durable immune memory to SARS-CoV-2 and variants of concern. Science **374**, abm0829 (2021). 34648302 10.1126/science.abm0829 PMC9284784

[27] D. Schaefer-Babajew , Antibody feedback regulates immune memory after SARS-CoV-2 mRNA vaccination. Nature **613**, 735–742 (2023). 36473496 10.1038/s41586-022-05609-w PMC9876794

[28] J. DeCuir , Effectiveness of monovalent mRNA COVID-19 vaccination in preventing COVID-19-associated invasive mechanical ventilation and death among immunocompetent adults during the omicron variant period—IVY Network, 19 U.S. States, February 1, 2022-January 31, 2023. Morb. Mortal. Wkly. Rep. **72**, 463–468 (2023). 10.15585/mmwr.mm7217a3 37104244

[29] I. D. Plumb , Effectiveness of COVID-19 mRNA vaccination in preventing COVID-19-associated hospitalization among adults with previous SARS-CoV-2 infection—United States, June 2021–February 2022. Morb. Mortal. Wkly. Rep. **71**, 549–555 (2022). 10.15585/mmwr.mm7115e2 PMC9020856 35421077

[30] A. S. Lauring , Clinical severity of, and effectiveness of mRNA vaccines against, COVID-19 from omicron, delta, and alpha SARS-CoV-2 variants in the United States: Prospective observational study. BMJ **376**, e069761 (2022). 35264324 10.1136/bmj-2021-069761 PMC8905308

[31] W. Li , Bats are natural reservoirs of SARS-like coronaviruses. Science **310**, 676–679 (2005). 16195424 10.1126/science.1118391

[32] M. Ruiz-Aravena , Ecology, evolution and spillover of coronaviruses from bats. Nat. Rev. Microbiol. **20**, 299–314 (2022). 34799704 10.1038/s41579-021-00652-2 PMC8603903

[33] A. A. Cohen , Mosaic nanoparticles elicit cross-reactive immune responses to zoonotic coronaviruses in mice. Science **371**, 735–741 (2021). 33436524 10.1126/science.abf6840 PMC7928838

[34] C. Fan , Neutralizing monoclonal antibodies elicited by mosaic RBD nanoparticles bind conserved sarbecovirus epitopes. Immunity **55**, 2419–2435.e2410 (2022). 36370711 10.1016/j.immuni.2022.10.019 PMC9606073

[35] D. B. Lee , Mosaic RBD nanoparticles induce intergenus cross-reactive antibodies and protect against SARS-CoV-2 challenge. Proc. Natl. Acad. Sci. U.S.A. **120**, e2208425120 (2023). 36669119 10.1073/pnas.2208425120 PMC9942827

[36] L. He , Presenting native-like trimeric HIV-1 antigens with self-assembling nanoparticles. Nat. Commun. **7**, 12041 (2016). 27349934 10.1038/ncomms12041 PMC4931238

[37] J. Jardine , Rational HIV immunogen design to target specific germline B cell receptors. Science **340**, 711–716 (2013). 23539181 10.1126/science.1234150 PMC3689846

[38] H. M. Yassine , Hemagglutinin-stem nanoparticles generate heterosubtypic influenza protection. Nat. Med. **21**, 1065–1070 (2015). 26301691 10.1038/nm.3927

[39] S. Boyoglu-Barnum , Quadrivalent influenza nanoparticle vaccines induce broad protection. Nature **592**, 623–628 (2021). 33762730 10.1038/s41586-021-03365-x PMC8269962

[40] M. Kanekiyo , Self-assembling influenza nanoparticle vaccines elicit broadly neutralizing H1N1 antibodies. Nature **499**, 102–106 (2013). 23698367 10.1038/nature12202 PMC8312026

[41] A. A. Cohen , Mosaic RBD nanoparticles protect against challenge by diverse sarbecoviruses in animal models. Science **377**, eabq0839 (2022). 35857620 10.1126/science.abq0839 PMC9273039

[42] M. A. G. Hoffmann , Escrt recruitment to SARS-CoV-2 spike induces virus-like particles that improve mRNA vaccines. Cell **186**, 2380–2391.e2389 (2023). 37146611 10.1016/j.cell.2023.04.024 PMC10121106

[43] A. A. Cohen , Mosaic sarbecovirus nanoparticles elicit cross-reactive responses in pre-vaccinated animals. Cell **187**, 5554–5571.e5519 (2024). 39197450 10.1016/j.cell.2024.07.052 PMC11460329

[44] C. Fan , Cross-reactive sarbecovirus antibodies induced by mosaic RBD nanoparticles. Proc. Natl. Acad. Sci. U.S.A. **122**, e2501637122 (2025). 40402246 10.1073/pnas.2501637122 PMC12130868

[45] K. S. Corbett , SARS-CoV-2 mRNA vaccine design enabled by prototype pathogen preparedness. Nature **586**, 567–571 (2020). 32756549 10.1038/s41586-020-2622-0 PMC7581537

[46] F. Zhang , Pan-sarbecovirus prophylaxis with human anti-ACE2 monoclonal antibodies. Nat. Microbiol. **8**, 1051–1063 (2023). 37188812 10.1038/s41564-023-01389-9 PMC10234812

[47] J. F. Drexler , Genomic characterization of severe acute respiratory syndrome-related coronavirus in European bats and classification of coronaviruses based on partial RNA-dependent RNA polymerase gene sequences. J. Virol. **84**, 11336–11349 (2010). 20686038 10.1128/JVI.00650-10 PMC2953168

[48] L. Yang , Novel SARS-like betacoronaviruses in bats, China, 2011. Emerg. Infect. Dis. **19**, 989–991 (2013). 23739658 10.3201/eid1906.121648 PMC3713832

[49] D. Planas , Reduced sensitivity of SARS-CoV-2 variant Delta to antibody neutralization. Nature **596**, 276–280 (2021). 34237773 10.1038/s41586-021-03777-9

[50] F. Schmidt , High genetic barrier to SARS-CoV-2 polyclonal neutralizing antibody escape. Nature **600**, 512–516 (2021). 34544114 10.1038/s41586-021-04005-0 PMC9241107

[51] C. O. Barnes , SARS-CoV-2 neutralizing antibody structures inform therapeutic strategies. Nature **588**, 682–687 (2020). 33045718 10.1038/s41586-020-2852-1 PMC8092461

[52] F. J. Weisel, G. V. Zuccarino-Catania, M. Chikina, M. J. Shlomchik, A temporal switch in the germinal center determines differential output of memory B and plasma cells. Immunity **44**, 116–130 (2016). 26795247 10.1016/j.immuni.2015.12.004 PMC4724390

[53] M. Akkaya, K. Kwak, S. K. Pierce, B cell memory: Building two walls of protection against pathogens. Nat. Rev. Immunol. **20**, 229–238 (2020). 31836872 10.1038/s41577-019-0244-2 PMC7223087

[54] M. Yuan , Structural basis of a shared antibody response to SARS-CoV-2. Science **369**, 1119–1123 (2020). 32661058 10.1126/science.abd2321 PMC7402627

[55] D. F. Robbiani , Convergent antibody responses to SARS-CoV-2 in convalescent individuals. Nature **584**, 437–442 (2020). 32555388 10.1038/s41586-020-2456-9 PMC7442695

[56] Z. Wang , Memory B cell development elicited by mRNA booster vaccinations in the elderly. J. Exp. Med. **220**, e20230668 (2023). 37368240 10.1084/jem.20230668 PMC10300432

[57] M. G. Brewer , Nanoparticles decorated with viral antigens are more immunogenic at low surface density. Vaccine **35**, 774–781 (2017). 28057386 10.1016/j.vaccine.2016.12.049 PMC5876043

[58] I. C. MacLennan , Extrafollicular antibody responses. Immunol. Rev. **194**, 8–18 (2003). 12846803 10.1034/j.1600-065x.2003.00058.x

[59] J. J. Mond, A. Lees, C. M. Snapper, T cell-independent antigens type 2. Annu. Rev. Immunol. **13**, 655–692 (1995). 7612238 10.1146/annurev.iy.13.040195.003255

[60] G. D. Victora, M. C. Nussenzweig, Germinal centers. Annu. Rev. Immunol. **40**, 413–442 (2022). 35113731 10.1146/annurev-immunol-120419-022408

[61] C. Viant , Germinal center-dependent and -independent memory B cells produced throughout the immune response. J. Exp. Med. **218**, e20202489 (2021). 34106207 10.1084/jem.20202489 PMC8193567

[62] J. S. Turner , SARS-CoV-2 mRNA vaccines induce persistent human germinal centre responses. Nature **596**, 109–113 (2021). 34182569 10.1038/s41586-021-03738-2 PMC8935394

[63] F. Schmidt , Measuring SARS-CoV-2 neutralizing antibody activity using pseudotyped and chimeric viruses. J. Exp. Med. **217**, e20201181 (2020). 32692348 10.1084/jem.20201181 PMC7372514

[64] F. Schmidt , Plasma neutralization of the SARS-CoV-2 omicron variant. N. Engl. J. Med. **386**, 599–601 (2022). 35030645 10.1056/NEJMc2119641 PMC8757565

[65] Z. Wang , Enhanced SARS-CoV-2 neutralization by dimeric IgA. Sci. Transl. Med. **13**, eabf1555 (2021). 33288661 10.1126/scitranslmed.abf1555 PMC7857415

[66] N. T. Gupta , Change-O: A toolkit for analyzing large-scale B cell immunoglobulin repertoire sequencing data. Bioinformatics **31**, 3356–3358 (2015). 26069265 10.1093/bioinformatics/btv359 PMC4793929

[67] C. Soto , High frequency of shared clonotypes in human B cell receptor repertoires. Nature **566**, 398–402 (2019). 30760926 10.1038/s41586-019-0934-8 PMC6949180

[68] Y. Guo, K. Chen, P. D. Kwong, L. Shapiro, Z. Sheng, cAb-Rep: A database of curated antibody repertoires for exploring antibody diversity and predicting antibody prevalence. Front. Immunol. **10**, 2365 (2019). 31649674 10.3389/fimmu.2019.02365 PMC6794461

