# Supplementary material for: Memory B cell development in response to mRNA SARS-CoV-2 and nanoparticle immunization in mice
Source: Proc Natl Acad Sci U S A. 2026 Jan 2;123(1):e2527869123. doi: 10.1073/pnas.2527869123 (PMC12773752; doi:10.1073/pnas.2527869123)
Supplement: Supplementary file 1 — Appendix 01 (PDF) [file pnas.2527869123.sapp.pdf]

Supporting Information for
Memory B cell Development in Response to mRNA SARS-CoV-2 and Nanoparticle Immunization in Mice

Authors: Marie Wiatr1, Zijun Wang1, Marie Canis2, Brianna Hernandez1, Anna Gazumyan1, Gabriela S. Silva Santos1, Sadman Shawraz2, Songhee Lee2, Paul D. Bieniasz2,3, Theodora Hatziioannou2, Frauke Muecksch2,4*, Michel C. Nussenzweig1,3*

1Laboratory of Molecular Immunology, The Rockefeller University; New York, NY 10065, USA.
2Laboratory of Retrovirology, The Rockefeller University; New York, NY 10065, USA.
3 Howard Hughes Medical Institute, The Rockefeller University, New York, NY, USA
4Department of Infectious Diseases, Virology, Medical Faculty Heidelberg, Heidelberg University; 69120 Heidelberg, Germany

* Corresponding authors:
Michel Nussenzweig
1230 York Avenue, New York, NY 10065
(212)327-8067
Email: nussen@rockefeller.edu,

Frauke Muecksch
Im Neuenheimer Feld 344, 69120 Heidelberg, Germany
+49(0)6221-5635643
Email: Frauke.muecksch@med.uni-heidelberg.de

This PDF file includes:

Figures S1 to S4
Tables S1 to S3

Figures

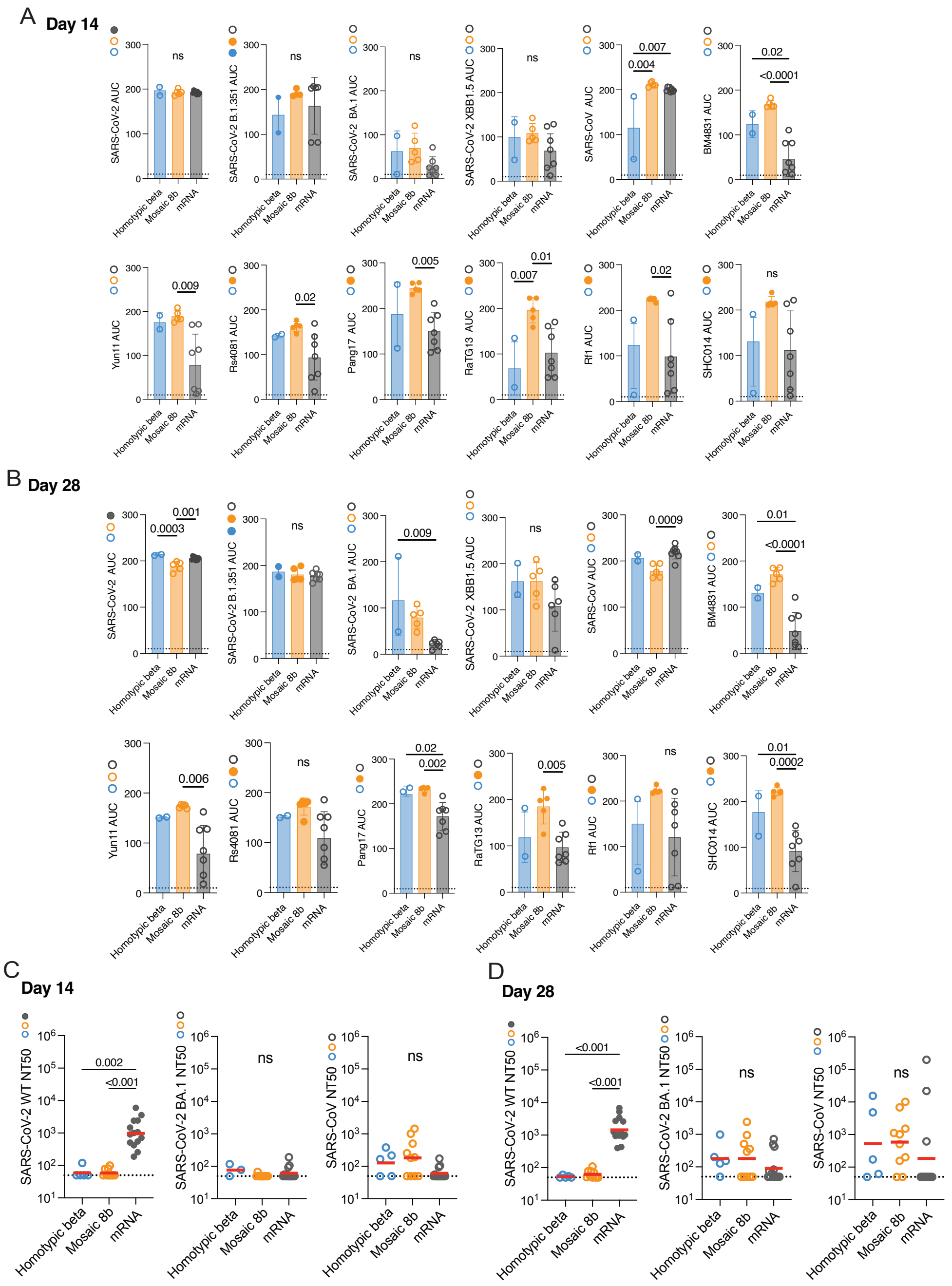

Fig. S1. Plasma binding and neutralizing activity. (A) Graphs show plasma IgG binding activity as normalized area under the curve (AUC) values measured by ELISA for; SARS-CoV-2, SARS-CoV-2 B.1.351, SARS-CoV-2 BA.1, SARS-CoV-2 XBB1.5, BM4831, Yun 11, Rs4081, Pang17, Ratg13, Rf1, and SHC014 RBDs on day 14 post prime. (B) Same as in (A) but measured on day 28. In (A) and (B) the dotted black line represents negative control. Each dot represents one animal. P values were calculated using an ANOVA test. (C and D) Plasma neutralizing titers for SARS-CoV-2, SARS-CoV-2 BA.1 and SARS-CoV. Plasma NT50s for homotypic beta (blue), mosaic 8b (orange) and mRNA (gray) measured on day 14 (C) and day 28 (D). Each dot represents one animal. The horizontal bar represents the geometric mean. All experiments were

performed at least 2X. P values were calculated using Kruskal-Wallis test followed by Dunn's multiple comparison test. Only P-values lower than 0.05 are shown.

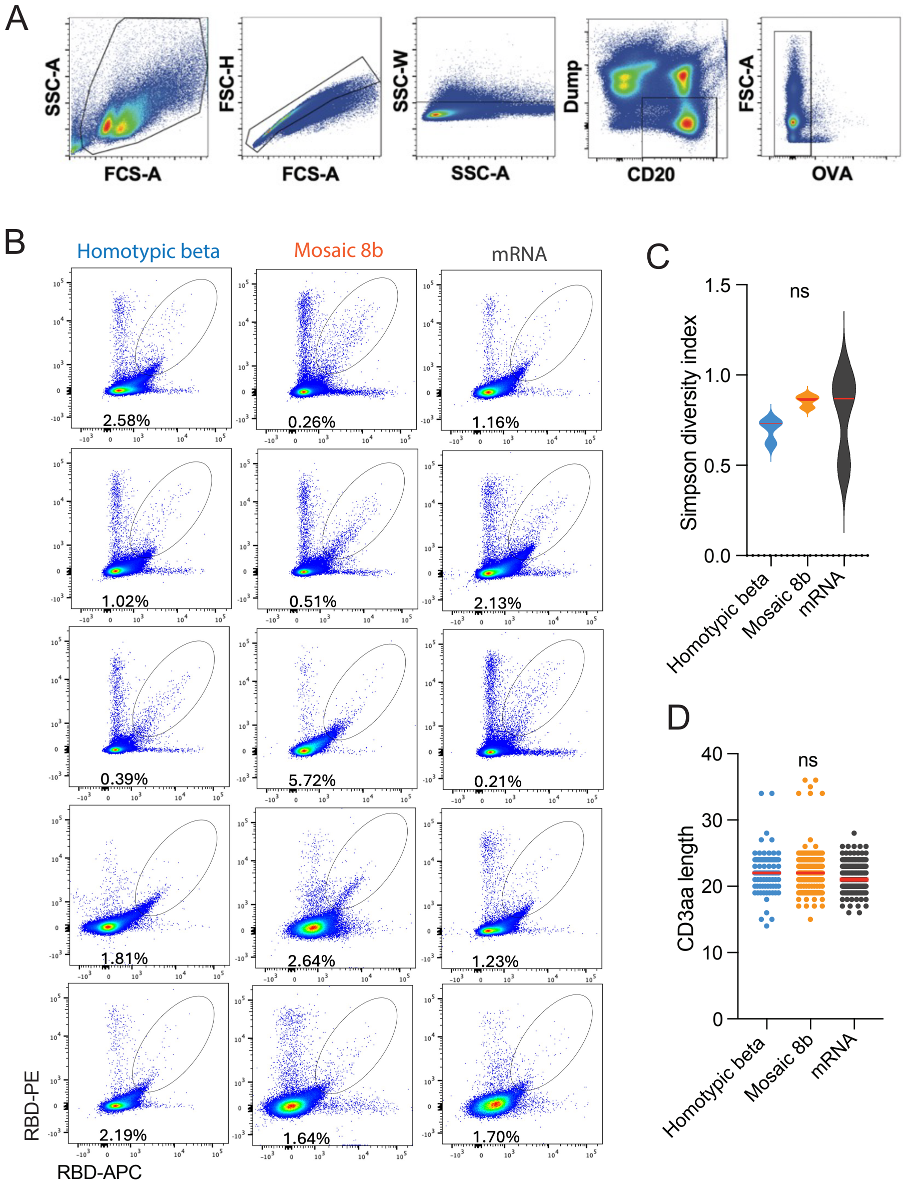

Fig. S2. Flow cytometry. (A) Gating strategy used for cell sorting. Doublets were excluded, CD20+ B cells selected and CD4+, CD8+, NKK1.1+, GR1+, OVA+ cells excluded. (B) Flow cytometry plots show the percentage of RBD+ B cells binding to phycoerythrin and allophycocyanin labeled RBDs in individual mouse lymph node on day 56 after immunization for homotypic beta-, mosaic 8b-, and mRNA-vaccinated mice. (C) Simpson diversity index for all antibodies in Fig. 2B and SI appendix; Table S2. (D) Combined CDR3 length for IgH and IgL for all antibodies in Fig. 2B and SI appendix; Table S2.

A

ID	Homotypic beta: SARS-CoV-2 WT	Beta	Omicron BA.1	XBB1.5	SARS-CoV	Epitope
752	1.2	0.3	2.6	1000.0	1000.0	II/III
754	2.6	0.3	7.4	1000.0	1000.0	II/III
755	5.4	0.3	21.5	885.7	1000.0	II/III
757	6.9	0.3	16.8	952.6	1000.0	II/III
770	7.5	0.7	7.1	1000.0	1000.0	II/III
758	9.0	1.0	21.4	1000.0	1000.0	II/III
759	9.5	0.8	11.7	1000.0	1000.0	II/III
750	10.7	0.5	22.8	27.5	141.2	II/III
760	12.5	1.3	10.3	1000.0	1000.0	II/III
751	35.0	1.4	52.4	1000.0	1000.0	II/III
676	140.3	1000.0	733.8	1000.0	150.3	UN
625	262.9	1000.0	744.5	456.0	1000.0	I
769	307.9	0.6	629.4	63.5	313.5	I/II/III/IV
673	343.9	1000.0	1000.0	641.7	1000.0	IV
675	373.8	30.4	1000.0	1000.0	1000.0	V
687	422.2	1000.0	1000.0	1000.0	1000.0	UN
762	855.7	439.1	1000.0	1000.0	44.1	I
624	1000.0	632.4	1000.0	1000.0	1000.0	UN
674	1000.0	880.6	1000.0	1000.0	1000.0	IV
626	1000.0	1000.0	1000.0	1000.0	1000.0	V
627	1000.0	1000.0	1000.0	1000.0	1000.0	I/II/III/IV
628	1000.0	1000.0	1000.0	1000.0	1000.0	UN
629	1000.0	1000.0	1000.0	1000.0	1000.0	I
630	1000.0	1000.0	1000.0	1000.0	1000.0	I/II/III/IV
670	1000.0	1000.0	1000.0	1000.0	1000.0	I/II
671	1000.0	1000.0	1000.0	1000.0	1000.0	I
672	1000.0	1000.0	1000.0	1000.0	1000.0	UN
677	1000.0	1000.0	1000.0	1000.0	1000.0	V
681	1000.0	1000.0	1000.0	1000.0	1000.0	UN
685	1000.0	1000.0	1000.0	1000.0	1000.0	UN
768	1000.0	1000.0	1000.0	1000.0	1000.0	I/III/IV

ID	Mosaic 8b: SARS-CoV-2 WT	Beta	Omicron BA.1	XBB1.5	SARS-CoV	Epitope
734	3.5	0.3	4.4	n.d.	n.d.	I
728	4.7	0.3	2.0	n.d.	750.0	I/II
740	17.2	0.3	144.3	0.3	16.8	I/V
716	54.8	0.3	2.9	138.2	1.1	I/V
711	64.3	0.6	2.5	240.8	1.1	I
745	74.1	178.3	57.8	61.3	1000.0	UN
721	119.1	0.3	1000.0	43.7	22.3	IV/V
713	135.2	0.3	6.3	8.7	4.8	I
735	189.1	0.3	603.7	2.4	62.4	I/V
718	208.1	30.8	1000.0	169.7	16.6	IV/V
702	213.0	0.3	656.6	2.1	13.9	I/V
739	233.6	0.3	783.0	13.6	122.6	I
729	340.9	50.3	212.5	1000.0	8.2	I
602	412.7	137.6	214.6	864.7	1000.0	UN
719	466.2	25.6	1000.0	1000.0	741.8	IV/V
710	651.6	2.3	18.9	1000.0	6.5	I/II/III/IV
703	687.7	0.3	746.0	61.7	733.6	UN
707	744.0	0.3	1000.0	839.0	1000.0	UN
404	826.3	1000.0	1000.0	1000.0	8.8	UN
725	848.7	2.3	1000.0	1000.0	164.0	IV/V
732	854.7	0.3	958.0	1000.0	1000.0	I/II/III/IV
619	905.0	415.2	1000.0	1000.0	6.8	I/IV
420	908.4	1000.0	1000.0	1000.0	n.d.	UN
705	1000.0	0.3	276.7	864.0	1000.0	UN
706	1000.0	0.3	923.1	1000.0	1000.0	UN
704	1000.0	0.3	1000.0	1000.0	1000.0	UN
731	1000.0	1.2	1000.0	1000.0	740.6	I
736	1000.0	2.5	1000.0	n.d.	n.d.	IV
724	1000.0	30.5	563.9	1000.0	140.5	IV/V
714	1000.0	37.0	68.3	1000.0	207.8	IV/V
726	1000.0	301.6	1000.0	1000.0	1000.0	III
742	1000.0	681.8	1000.0	n.d.	1000.0	I/II/III
401	1000.0	855.3	1000.0	1000.0	1000.0	I/IV
744	1000.0	1000.0	1000.0	810.1	1000.0	UN
730	1000.0	1000.0	1000.0	1000.0	20.3	I
400	1000.0	1000.0	1000.0	1000.0	1000.0	I/IV
403	1000.0	1000.0	1000.0	1000.0	1000.0	IV
407	1000.0	1000.0	1000.0	1000.0	1000.0	UN
601	1000.0	1000.0	1000.0	1000.0	1000.0	I
606	1000.0	1000.0	1000.0	1000.0	1000.0	I
620	1000.0	1000.0	1000.0	1000.0	1000.0	I/IV
727	1000.0	1000.0	1000.0	1000.0	1000.0	I
607	1000.0	1000.0	1000.0	1000.0	n.d.	IV
746	1000.0	1000.0	1000.0	1000.0	1000.0	I/IV
733	1000.0	30.9	1000.0	947.1	1000.0	V
723	1000.0	n.d.	1000.0	612.1	817.8	I/IV
722	1000.0	1000.0	1000.0	1000.0	1000.0	V

ID	mRNA: SARS-CoV-2 WT	Beta	Omicron BA.1	XBB1.5	SARS-CoV	Epitope
900	0.5	0.3	780.5	1000.0	1000.0	III
512	0.6	0.3	1000.0	1000.0	1000.0	III
323	0.8	0.3	1000.0	1000.0	1000.0	UN
332	1.1	331.2	1000.0	1000.0	1000.0	III
327	1.1	0.3	1000.0	1000.0	1000.0	UN
330	1.2	0.3	1000.0	1000.0	1000.0	III
329	1.5	0.3	1000.0	1000.0	1000.0	UN
331	1.5	0.3	1000.0	1000.0	1000.0	III
335	2.1	0.3	1000.0	1000.0	1000.0	III
328	2.3	0.3	274.0	1000.0	1000.0	UN
909	8.6	1.7	n.d.	48.3	44.9	I
315	9.1	1000.0	1000.0	1000.0	1000.0	UN
326	9.4	0.3	1000.0	1000.0	1000.0	UN
316	16.3	1000.0	1000.0	1000.0	1000.0	UN
908	21.5	1000.0	1000.0	1000.0	1000.0	UN
913	36.6	2.5	42.6	93.7	98.3	IV/V
513	38.4	1000.0	873.3	1000.0	797.1	II
912	150.9	51.7	63.1	74.7	1000.0	V
917	173.4	1000.0	1000.0	1000.0	1000.0	I/II
341	178.8	64.7	204.9	1000.0	1000.0	V
349	203.6	43.7	157.0	219.2	1000.0	V
344	248.0	72.2	192.7	113.0	1000.0	V
804	349.4	700.9	1000.0	1000.0	1000.0	IV
340	549.5	91.1	142.0	314.9	1000.0	V
802	560.4	1000.0	1000.0	1000.0	1000.0	IV
343	594.6	244.2	91.1	426.6	1000.0	V
342	676.5	97.6	154.1	408.7	1000.0	V
348	727.9	88.5	104.6	89.4	1000.0	V
916	742.2	1000.0	1000.0	1000.0	1000.0	I
920	758.1	183.8	1000.0	1000.0	1000.0	IV
347	787.4	95.3	148.6	72.0	1000.0	V
806	864.3	1000.0	1000.0	1000.0	1000.0	IV
346	884.7	104.2	1000.0	1000.0	100.0	IV
907	911.2	1000.0	1000.0	1000.0	1000.0	IV
915	927.9	1000.0	1000.0	1000.0	1000.0	IV
805	1000.0	41.5	1000.0	1000.0	1000.0	IV
320	1000.0	186.6	1000.0	1000.0	1000.0	V
801	1000.0	661.2	1000.0	1000.0	1000.0	IV
345	1000.0	1000.0	1000.0	1000.0	200.1	IV
300	1000.0	1000.0	1000.0	1000.0	1000.0	IV
301	1000.0	1000.0	1000.0	1000.0	1000.0	IV
302	1000.0	1000.0	1000.0	1000.0	1000.0	IV
303	1000.0	1000.0	1000.0	1000.0	1000.0	IV
304	1000.0	1000.0	1000.0	1000.0	1000.0	IV
306	1000.0	1000.0	1000.0	1000.0	1000.0	UN
307	1000.0	1000.0	1000.0	1000.0	1000.0	UN
308	1000.0	1000.0	1000.0	1000.0	1000.0	UN
309	1000.0	1000.0	1000.0	1000.0	1000.0	UN
313	1000.0	1000.0	1000.0	1000.0	1000.0	IV
317	1000.0	1000.0	1000.0	1000.0	1000.0	UN
318	1000.0	1000.0	1000.0	1000.0	1000.0	UN
319	1000.0	1000.0	1000.0	1000.0	1000.0	V
321	1000.0	1000.0	1000.0	1000.0	1000.0	IV
352	1000.0	1000.0	1000.0	1000.0	1000.0	IV
355	1000.0	1000.0	1000.0	1000.0	1000.0	UN
356	1000.0	1000.0	1000.0	1000.0	1000.0	V
357	1000.0	1000.0	1000.0	1000.0	1000.0	UN
358	1000.0	1000.0	1000.0	1000.0	1000.0	I
819	1000.0	1000.0	1000.0	1000.0	1000.0	I/II/III/IV
820	1000.0	1000.0	1000.0	1000.0	1000.0	I/II/III/IV
902	1000.0	971.7	1000.0	1000.0	1000.0	UN
911	1000.0	1000.0	1000.0	1000.0	1000.0	I
914	1000.0	1000.0	1000.0	1000.0	1000.0	II
904	n.d.	10.6	n.d.	n.d.	n.d.	V
514	n.d.	689.9	1000.0	1000.0	1000.0	UN
800	n.d.	822.5	1000.0	787.3	722.9	IV

B

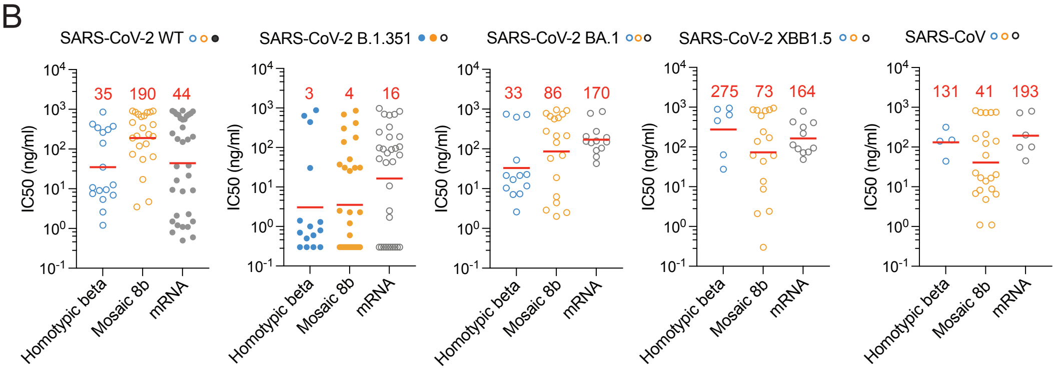

Fig. S3. Neutralization activity of monoclonal antibodies. (A) Tables showing the IC50 (ng/ml) for each monoclonal antibody against the 5 tested pseudoviruses, SARS-CoV-2, SARS-CoV-2 B.1.351, SARS-CoV-2 BA.1, SARS-CoV-2 XBB1.5, and SARS-CoV. Color coding is white=most potent neutralizers [IC50<10ng/ml], red=no neutralization [IC50>=1000ng/ml]. (B) SARS-CoV-2, SARS-CoV-2 B.1.351, SARS-CoV-2 BA.1, SARS-CoV-2 XBB1.5 and SARS-CoV pseudovirus neutralization activity for antibodies with a detectable IC50 values in ng/ml for the indicated pseudoviruses. The red line shows the geometric mean indicated in red. Statistical significance was determined using the Kruskal-Wallis test with subsequent Dunn's multiple-comparisons test.

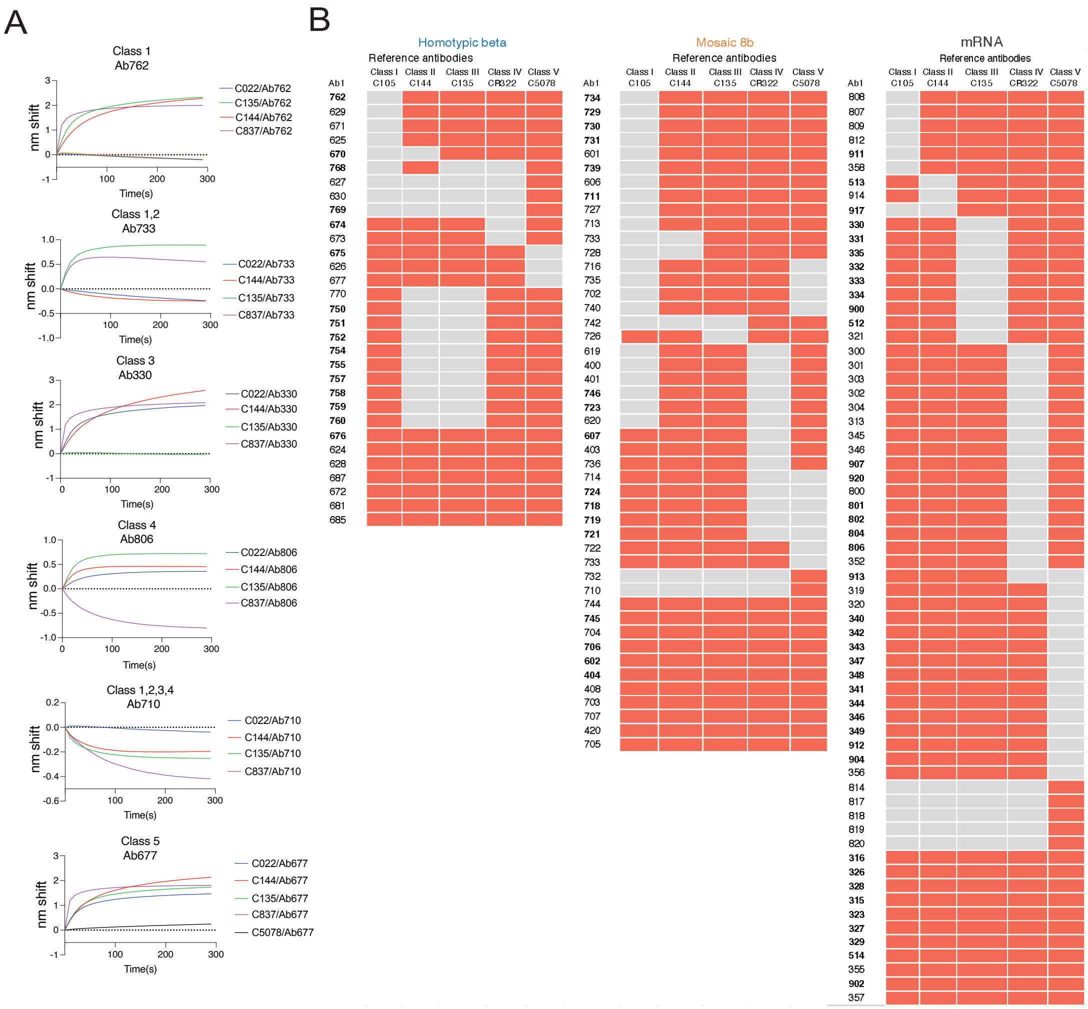

Fig. S4. Biolayer interferometry determination of epitope binding. (A) BLI curves showing interference of pre-formed antibody-RBD complexes with reference antibodies belonging to class 1 (C022, blue), class 2 (C144, yellow), class 3 (C135, orange), class 4 (C837, green) and class 5 (C5078, purple). (B) Tables display the BLI-mapped class of monoclonal antibodies for each immunization (homotypic beta, mosaic 8b and mRNA). Reference class-indicating antibodies are listed on the top. Grey indicates the class in which tested monoclonal antibodies belong, red the exclusion of the class. Bold antibodies are neutralizers.

Tables

	SARS-CoV-2	SARS-CoV-2 BA.1	SARS-CoV-2 B1.351	SARS-CoV-2 XBB1.5	SARS-CoV-2 B1.617	RaTG13	SHC014	Rs4081	Rf1	WIV1	Yun11	Pang17	SARS-CoV	BM4831	RmYN02
SARS-CoV-2		93.3	98.7	90.1	99.1	90.1	76.2	63.7	64.6	75.3	63.7	86.5	73.1	70.1	63.2
SARS-CoV-2 BA.1	93.3		94.2	94.2	93.3	87.4	75.3	62.8	63.7	72.2	61.9	83.0	70.9	68.3	61.9
SARS-CoV-2 B1.351	98.7	94.2		91.0	97.8	89.7	76.2	63.7	64.6	74.9	63.7	86.5	73.1	70.1	63.2
SARS-CoV-2 XBB1.5	90.1	94.2	91.0		90.1	86.1	74.9	61.0	62.3	71.3	60.1	82.1	70.0	67.4	60.1
SARS-CoV-2 B1.617	99.1	93.3	97.8	90.1		90.1	75.8	63.7	64.6	75.8	63.7	85.7	73.5	70.1	63.2
RaTG13	90.1	87.4	89.7	86.1	90.1		75.8	63.7	63.7	76.2	63.2	87.9	74.9	68.8	62.8
SHC014	76.2	75.3	76.2	74.9	75.8	75.8		63.1	64.4	84.2	64.4	75.8	82.0	74.9	64.0
Rs4081	63.7	62.8	63.7	61.0	63.7	63.7	63.1		89.7	64.9	92.6	65.0	63.5	62.1	88.7
Rf1	64.6	63.7	64.6	62.3	64.6	63.7	64.4	89.7		65.3	89.2	65.0	64.0	64.4	90.2
WIV1	75.3	72.2	74.9	71.3	75.8	76.2	84.2	64.9	65.3		66.2	74.9	94.6	73.5	64.9
Yun11	63.7	61.9	63.7	60.1	63.7	63.2	64.4	92.6	89.2	66.2		64.1	64.9	62.1	88.7
Pang17	86.5	83.0	86.5	82.1	85.7	87.9	75.8	65.0	65.0	74.9	64.1		74.9	68.3	63.2
SARS-CoV	73.1	70.9	73.1	70.0	73.5	74.9	82.0	63.5	64.0	94.6	64.9	74.9		72.2	63.5
BM4831	70.1	68.3	70.1	67.4	70.1	68.8	74.9	62.1	64.4	73.5	62.1	68.3	72.2		62.1
RmYN02	63.2	61.9	63.2	60.1	63.2	62.8	64.0	88.7	90.2	64.9	88.7	63.2	63.5	62.1	

Table S1. Percentage of amino acids similarity from the different Sarbecovirus RBD protein (receptor binding domain); RaTG13, SARS-CoV-2 B.1.351, Rs4081, SHC014, W1V1, BM4831, Rf1, SARS-CoV-2 B.1.617, SARS-CoV-2, SARS-CoV-2 BA.1, Yun11, SARS-CoV-2 XBB1.5, Pang17, RmYN02 and SARS-CoV. Color coding is white=low similarity between two RBDs, red= high similarity between two RBDs.

Table S2 and Table S3 can be found at https://doi.org/10.5281/zenodo.17515419.

Table S2. Sequences of anti-SARS-CoV-2 RBD IgG antibodies. Sequences derived from each mouse are shown separately for homotypic beta, mosaic 8b, and mRNA. Clonally related sequences (same V and J genes for heavy and light chains and similar CDR3) share the same background color; white background indicates singlets.

Table S3. Sequences of monoclonal IgG antibodies produced. First column of the table is the name of the antibody produced followed by the nucleotide sequence of the paired heavy in blue and light chain in white.
